# STAT3 and SPI1, may lead to the immune system dysregulation and heterotopic ossification in ankylosing spondylitis

**DOI:** 10.1186/s12865-022-00476-6

**Published:** 2022-01-22

**Authors:** Tuo Liang, Jiarui Chen, GuoYong Xu, Zide Zhang, Jiang Xue, Haopeng Zeng, Jie Jiang, Tianyou Chen, Zhaojie Qin, Hao Li, Zhen Ye, Yunfeng Nie, Xinli Zhan, Chong Liu

**Affiliations:** 1 Spine and Osteopathy Ward, The First Affiliated Hospital of Guangxi Medical University, No.6 Shuangyong Road, Nanning, Guangxi People’s Republic of China; 2 Guangxi Medical University, No.22 Shuangyong Road, Nanning, Guangxi People’s Republic of China

**Keywords:** Ankylosing spondylitis, Th1 cells, NKT cells, STAT3, SPI1, Biomarkers

## Abstract

**Objective:**

This study was aimed to identify the biomarkers for diagnosis and reveal the immune microenvironment changes in ankylosing spondylitis (AS).

**Methods:**

GSE73754 was downloaded for the co-expression network construction and immune cell analyses. Flow cytometric analysis was performed to validate the results of bioinformatics analysis. Gene set enrichment analysis (GSEA) was performed to investigate the potential biological characteristic between different phenotypes. Pearson correlation analysis between the hub genes and the xCell score of immune cell types was performed.

**Results:**

Signal transducer and activator of transcription 3 (STAT3) and Spi-1 proto-oncogene (SPI1) was identified as the hub genes in the datasets GSE73754. And the t-test showed that the expression level of STAT3 and SPI1 in the GSE73754 was significantly higher in AS and human leukocyte antigen (HLA)-B27(+) groups. Flow cytometric analysis showed that natural killer T cells (NKT) cells were upregulated, while Th1 cells were down-regulated in AS, which was consistent with the results obtained from bioinformatics analysis. STAT3 and SPI1 was correlated with the NKT cells and Th1 cells.

**Conclusion:**

STAT3 and SPI1 may be a key cytokine receptor in disease progression in AS.

**Supplementary Information:**

The online version contains supplementary material available at 10.1186/s12865-022-00476-6.

## Introduction

Ankylosing spondylitis (AS), a chronic inflammatory autoimmune disease, is a global disease diagnosed by millions of people every year, and it mainly occurred in young adult males [1]. AS mostly involves sacroiliac joints and axial skeleton and finally resulting in impairments of structure and function [2]. AS is caused by the loss of tolerance to autoantigens and autoantibodies' development because of the effectors of innate and adaptive immunity, which ultimately results in tissue inflammation and damage [3]. AS's pathogenesis is multifactorial, involving multiple cell types, cellular processes, and molecular mediators and regulators [4, 5]. Studies show that the major histocompatibility complex region (MHC) is closely related to AS, and about 95% AS patients are born with a gene named HLA-B27 [6]. However, HLA-B27 predisposition fails to fully explain the cause of the disease, and this leads to a strong effort to identify additional predisposing factors.

Dysregulation of the immune cells conferred risk for the pathogenesis of human autoimmune diseases including AS [7]. The balance between 1 helper T (Th1) and Th2 cells regulates the choice between inflammatory and antibody-mediated immune responses [8]. Those antibodies contain the autoantibody. The balance between Th1 and Th2 cells may also related to AS. A previous study suggested that Th17 cells and NKT cells secrete IL-17, which is a crucial pathologic cytokine in AS pathogenesis [9]. However, which genes lead to immune system dysregulation in AS also remained unknown. Therefore, there is an urgent need to predict the pathogenesis of AS.

Heterotopic ossification is an important factor in spinal fusion in AS. Bone formation requires critical osteogenic molecules and pathways, inflammation-induced activation of osteogenic signaling pathways is considered a logical link between inflammation and new bone formation. Wnt, MAPK, JAK/STAT signaling pathways have been previously investigated and shown to be involved in spinal fusion in patients with AS [10–12]. There are many cytokines being identified that regulate osteoblasts/osteoclasts differentiation. Moreover, immune cells can secrete cytokines which play a key role in osteoblasts/osteoclasts differentiation. Thus, in the present study, we utilized several bioinformatics approaches to further investigate the molecule mechanism of AS. How the key genes affect immune cells that leads to heterotopic ossification in AS. That may help develop new therapies that treat AS by exploring the complex mechanisms of immune responses.

In the present study, STAT3 and SPI1, which were identified as hub genes, could work as biomarkers for AS diagnosis. Secondly, we demonstrated STAT3 and SPI1 were involved in the activation, proliferation, and differentiation of Th1 cells and NKT cells in AS. STAT3 and SPI1 were involved in heterotopic ossification in AS. Additionally, the balance between Th1 cells and NKT cells may related to the osteogenesis and osteolysis in AS. STAT3 and SPI1 may be the key genes that lead to the dysregulation of the immune system and contributing to the pathogenesis of AS.

## Results

### Identification of stable modules associated with AS

**Fig. 1:**
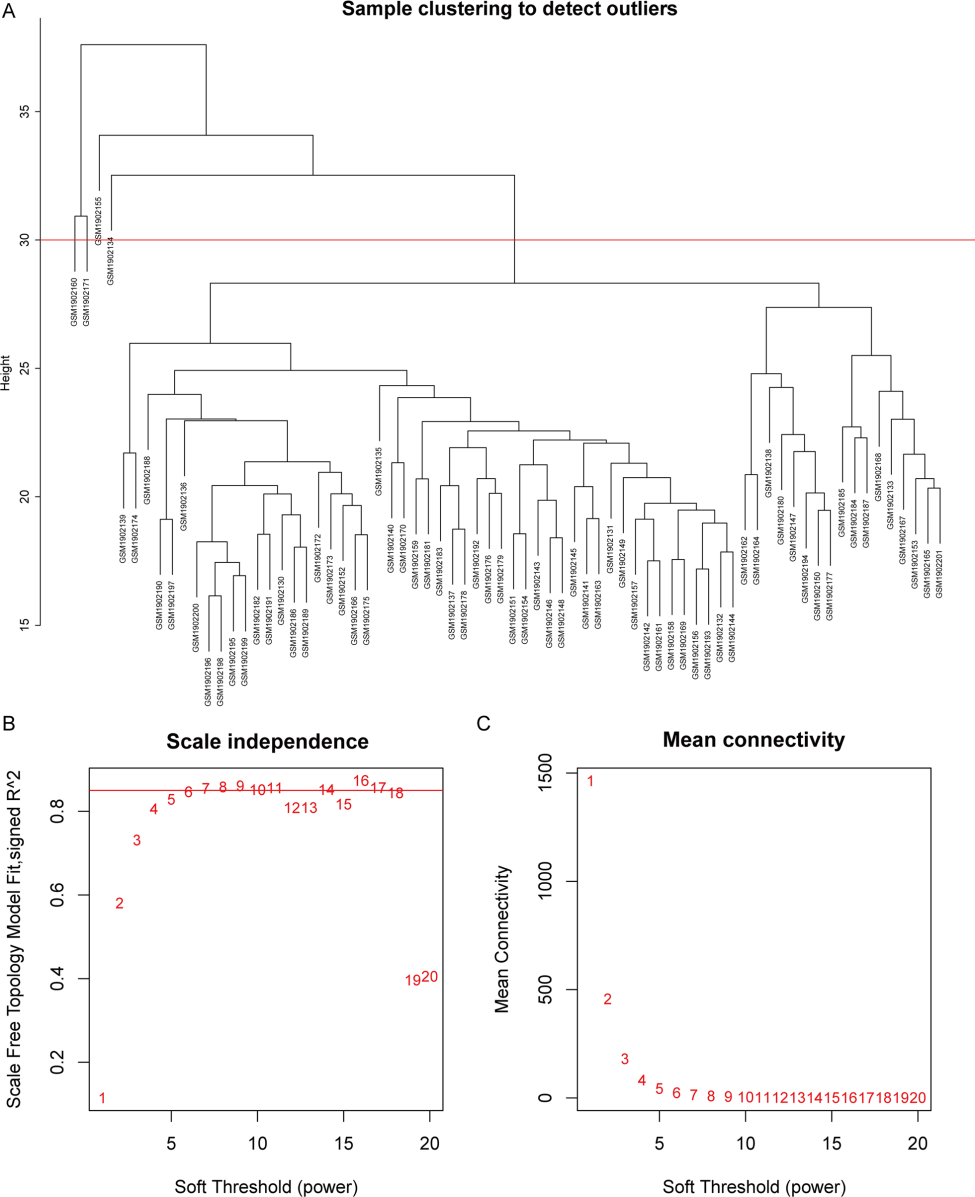
Weighted gene co-expression network analysis. **A** Clustering of samples to detect outliers in GSE73754. **B** The scale-free fit index as a function of the soft-threshold power. **C** The mean connectivity as a function of the soft-threshold power

**Fig. 2:**
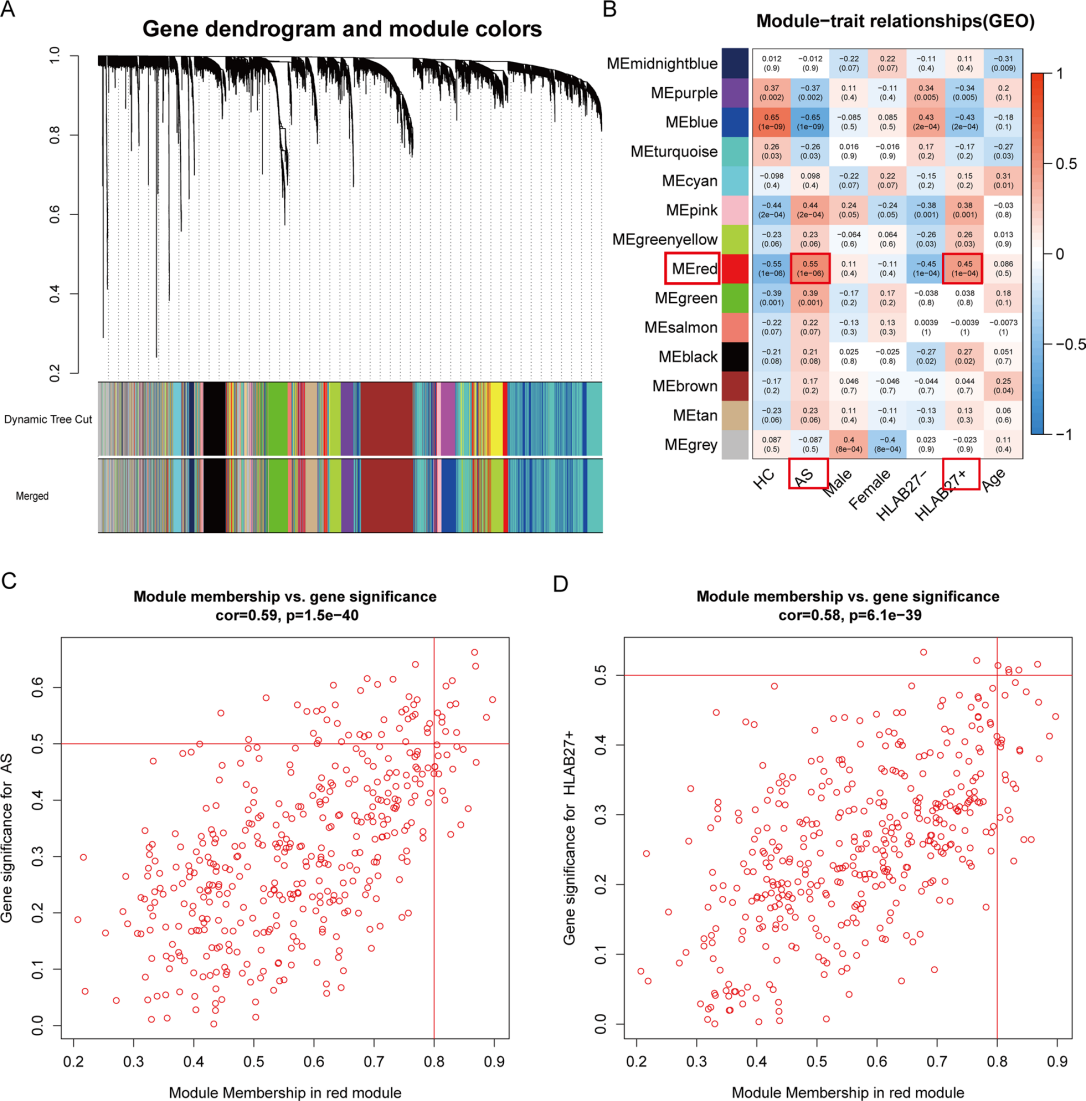
The results of WGCNA. **A** Cluster dendrogram based on the dynamic tree. Different dendrogram colors represent various modules. **B** Heatmap of the correlation between module eigengenes and different clinical characteristics of AS. **C** A scatterplot of gene significance for AS vs. module membership in the red module. **D** A scatterplot of gene significance for HLA-B27(+) versus module membership in the red module

WGCNA was performed on the 7395 genes of 72 samples in GSE73754. A total of 68 samples remained for subsequent analysis after four outlier samples were removed (Fig. 1A). The connectivity between genes in the gene network met a scale-free network distribution with a soft threshold power of β = 6 (scale-free R2 = 0.85) (Fig. 1B, C). After merging similar clusters, fourteen modules that contained groups of genes with shown in similar patterns of connection strengths with other genes were identified (Fig. 2A). We found significant correlation between HLA-B27 (+) and “red module” in patients with AS (Fig. 2B). In addition, scatterplots of gene significance versus module membership in the red module showed that they were highly correlated (Fig. 2C, D).

### Enrichment analyses and diagnosis ability of the hub genes

**Fig. 3:**
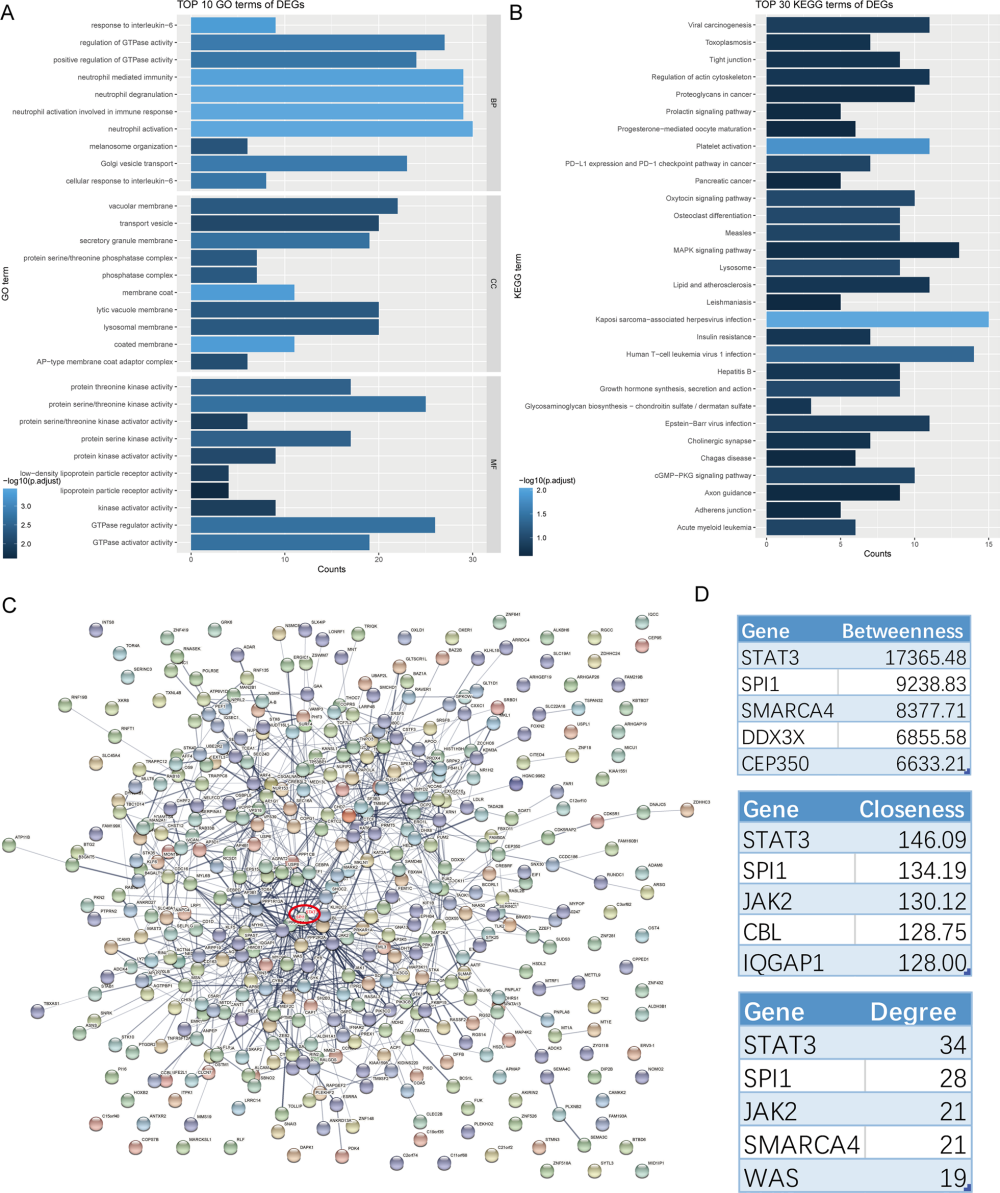
The GO\KEGG analysis results and PPI network for the DEGs in the red module in the GSE73754. The GO (**A**) and KEGG (**B**) analysis results for the DEGs in the red module in the GSE73754. **C** PPI network for the DEGs in the red module in the GSE73754. The red circle showed the position of STAT3 and SPI1. **D** The top five genes were arranged in descending order of “Degree”, “Betweenness”, and “Closeness” respectively

A total of 416 genes in red module were performed enrichment analyses. GO analysis of the genes in red module in the GSE73754 illustrated that the main biological processes (BP), cellular components (CC) and molecular functions (MF) were all related to immune response (Fig. 3A). KEGG pathway analysis to the genes in red module in the GSE73754 suggested that the genes were particularly involved in immune response and immune cell differentiation (Fig. 3B).

**Fig. 4:**
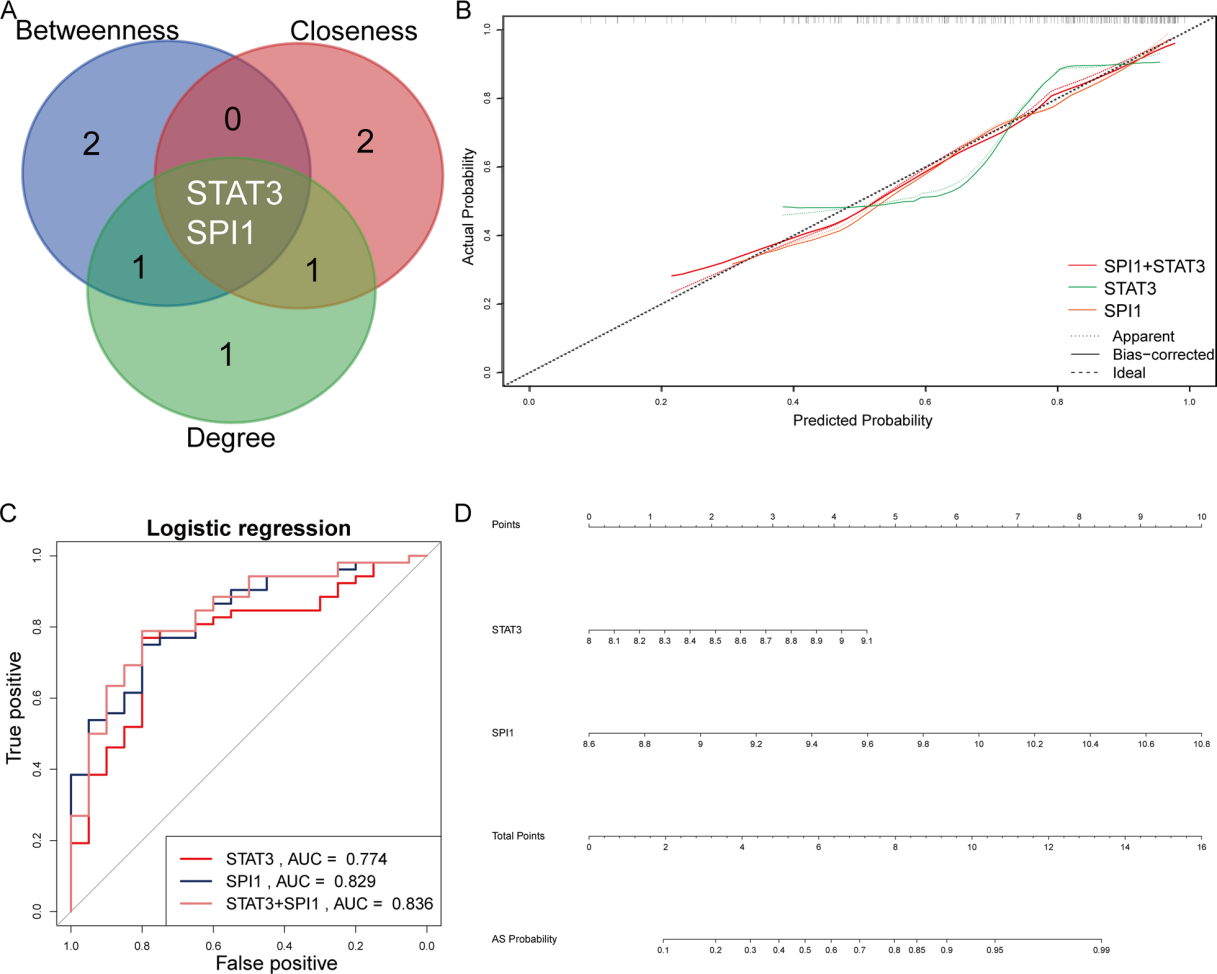
Hub genes identification. **A** The Venn diagram showed the hub genes in the GSE73754. **B** The calibration plots of different model for diagnosis. **C** ROC curves of hub genes’ diagnostic ability. **D** Nomogram for predicting the probability of AS

In order to screen out the hub genes from the genes in red module, all the genes in red module were uploaded to the STRING for further analysis. The PPI network were showed in Fig. 3C. Figure 3D showed the top five genes which were arranged in descending order of “Degree”, “Betweenness”, and “Closeness” respectively. STAT3 and SPI1 were identified as hub genes in the GSE73754 (Fig. 4A). Calibration curves indicated a satisfactory agreement between prediction and actual probabilities (Fig. 4B), prediction efficiency was much better especially when STAT3 and SPI1 combined. Furthermore, the AUCs of the ROC curves were 0.774 for STAT3, 0.829 for SPI1, and 0.836 for both combined (Fig. 4C). A nomogram (Fig. 4D) based on STAT3 and SPI1 was constructed for advancing clinical diagnosis of AS.

### Relationship between STAT3 and SPI1 and clinical traits

**Fig. 5:**
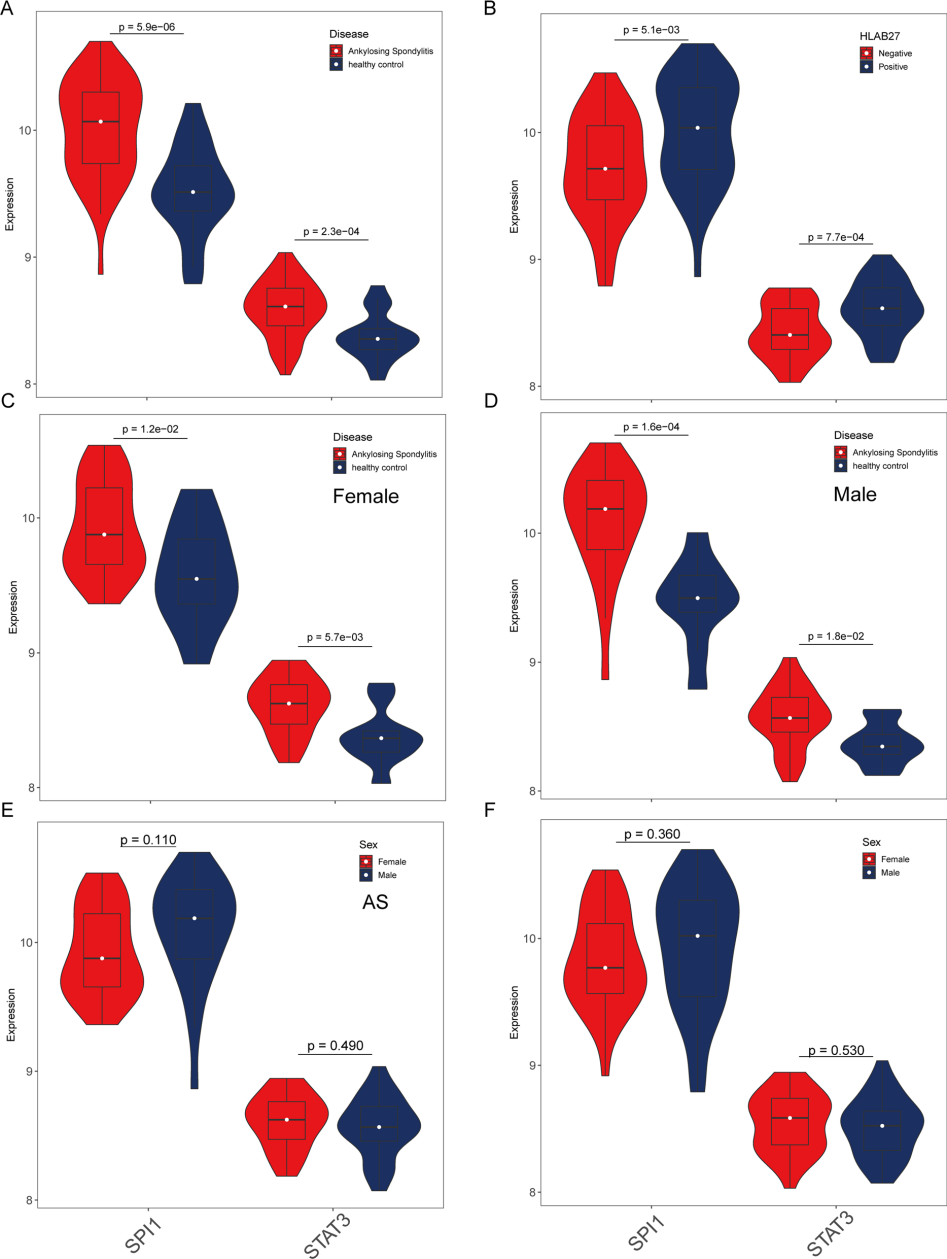
The correlation of STAT3 and SPI1 expression with clinical characteristics. **A** The expression of STAT3 and SPI1 between AS and HCs. **B** The expression of STAT3 and SPI1 between HLA-B27 (−) and HLA-B27 (+). **C** The expression of STAT3 and SPI1 between AS and HCs in female patients. **D** The expression of STAT3 and SPI1 between AS and HCs in male patients. **E** The expression of STAT3 and SPI1 between male and female in AS patients. **F** The expression of STAT3 and SPI1 between male and female in all patients

STAT3 and SPI1 were up-regulated in the AS group (Fig. 5A). In addition, STAT3 and SPI1 were also up-regulated in the HLA-B27(+) group (Fig. 5B). Both STAT3 and SPI1 expression were up-regulated in both male and female patients with AS (Fig. 5C, D). However, there were no significant difference between male and female patients with AS (Fig. 5E, F).

### Correlation between STAT3 and SPI1 and immune cells

**Fig. 6:**
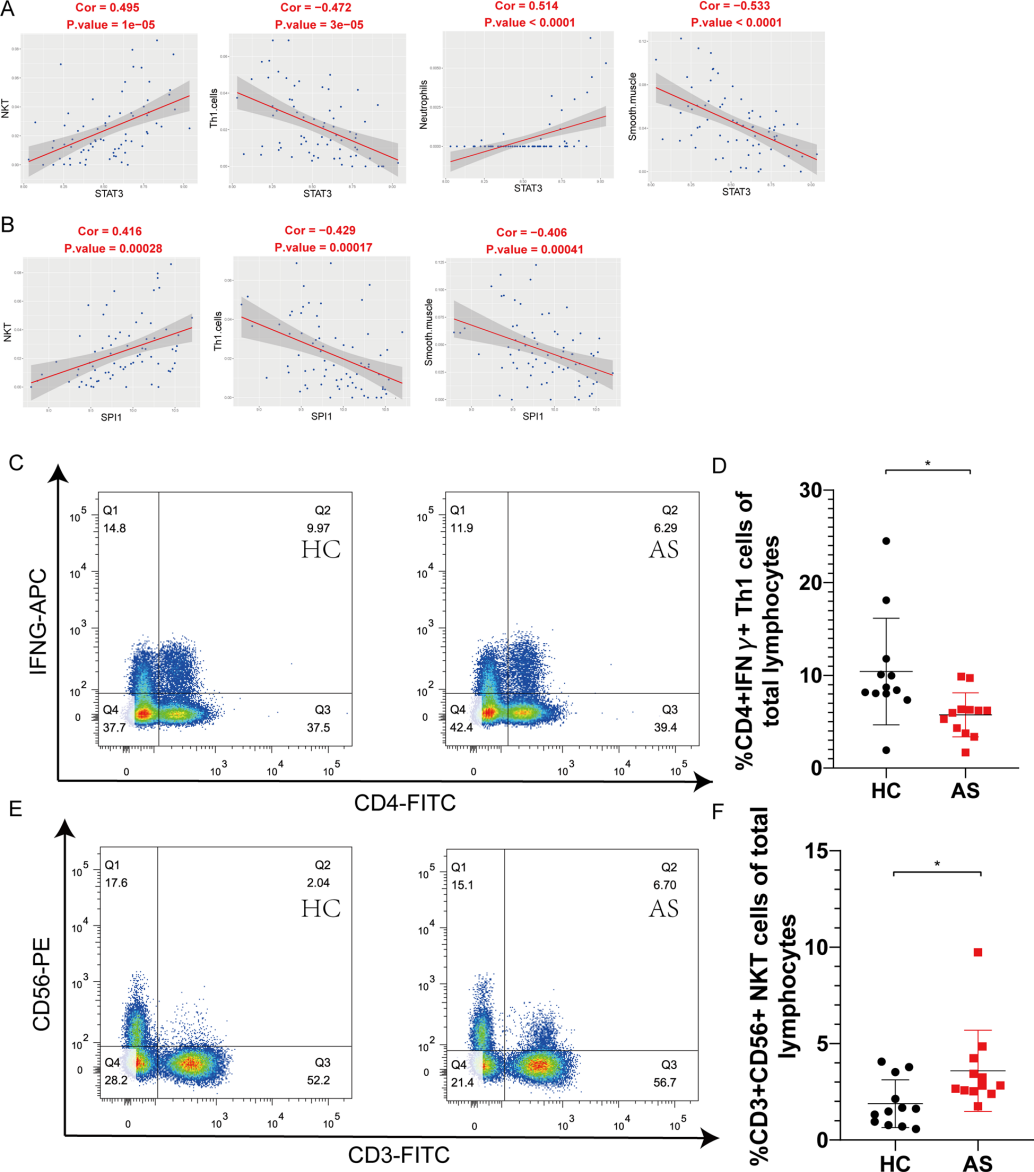
The correlation of STAT3 and SPI1 expression with xCell score. **A** The correlation of the expression of STAT3 with xCell score in GSE73754. **B** The correlation of the expression of SPI1 with xCell score in GSE73754. **C**, **E** Dot plots of a representative AS patient and HC. **D** Percentage of Th1 cells (% of CD4 + IFNγ + T cells). **F** Percentage of NKT cells (% of CD3 + CD56 + T cells)

The cell types potentially involved in AS were determined using xCell, which generates cell type enrichment scores using two datasets' gene expression data. The cell types that were significantly different between AS and HCs in the GSE73754 dataset are shown in Additional file 1: Fig. S1. We evaluate the correlation between the xCell scores of immune cells and the expression level of STAT3 and SPI1. STAT3 and SPI1 were significantly correlated with the xCell scores of NKT cells, Th1 cells, and smooth muscle in the GSE73754 (Fig. 6A, B). Flow cytometry was performed to validate the difference between NKT cells and Th1 cells between AS and HCs. NKT cells were upregulated (Fig. 6C, D), while Th1 cells were downregulated in AS (Fig. 6E, F), which was consistent with the results obtained from bioinformatics analysis.

### GSEA analysis

**Fig. 7:**
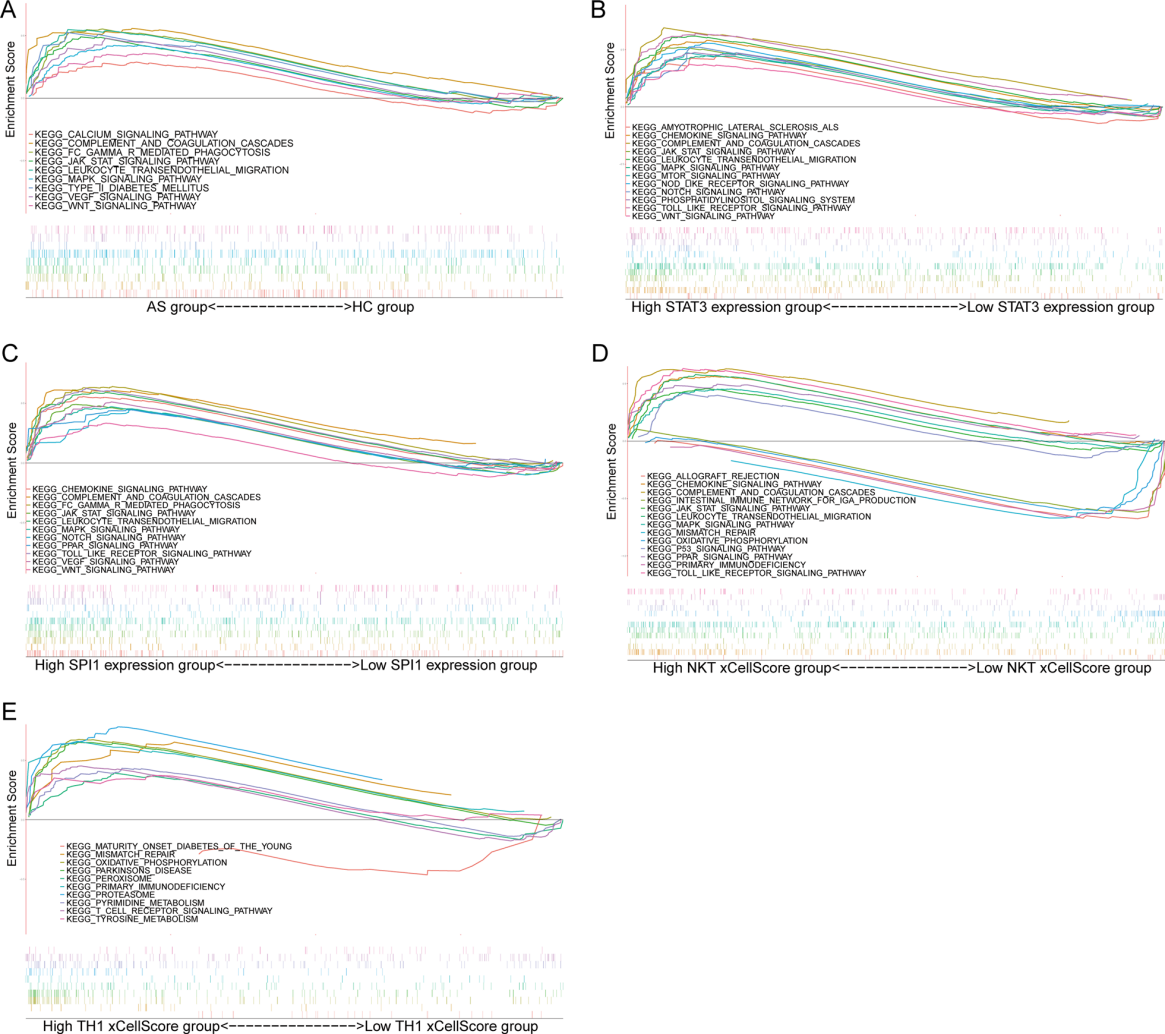
GSEA analysis. **A** GSEA in the KEGG gene set between AS (n = 52) and HCs (n = 20) group in GSE73754. **B**–**E** GSEA results of the KEGG backgroung between high (n = 36) and low (n = 36) of gene expression and xCell score in GSE73754

GSEA results on KEGG pathways revealed that most of the gene sets focused on immune-related pathways. A significant number of genes were enriched in pathways related to immunity among others. KEGG results showed that a majority of the genes were enriched in Fc-gamma R mediated phagocytosis, JAK/STAT signaling pathway, MAPK signaling pathway, VEGF receptor signaling pathway, and Wnt receptor signaling pathway in the AS group (Fig. 7A). KEGG analyses to the high STAT3/SPI1 expression and high-NKT xCell score showed the similar results in the AS group (Fig. 7B–D). KEGG results showed that most of the genes were enriched in oxidative phosphorylation, primary-immunodeficiency, proteasome, T-cell receptor signaling pathway in the high-Th1 xCell score group (Fig. 7E). The results provide clues into the in-depth biomechanism of AS and maybe a foundation for the subsequent application of AS immunotherapy.

## Discussion

It has been proved that AS is a chronic autoimmunity disease characterized by inflammation in the spine and sacroiliac joints resulting in initial bone and joint erosion [16]. As we know, the onset of AS suffers from the relationship between the host genetics, the intestinal microbiome, and the immune response [17]. Autoimmune dysregulation leads to tissue inflammation and damage because of the loss of tolerance to autoantigens and autoantibodies' development. Hence, developing a better understanding of the mechanism of autoimmune dysregulation and its relationship with the key genes in AS is paramount.

In the present study, STAT3 and SPI1 was identified as the hub genes, which was up-regulated in AS. STAT3 is a member of the signal transducer and activator of transcription protein family, which are phosphorylated by the receptor associated kinases, and then form homo- or heterodimers that translocate to the cell nucleus where they act as transcription activators [18]. STAT3 mediates the expression of a variety of genes in response to cell stimuli, and thus plays a key role in many cellular processes such as cell growth, apoptosis, activation and differentiation [19]. A previous study reported that the expression of STAT3 and proteins were elevated during active RA synovitis [20]. STAT3 had also been reported associated with the pathogenesis of AS. Within the osteoblast lineage, STAT3 signaling promotes bone formation in normal physiology and in response to mechanical loading through direct signaling in osteocytes [21]. The GSEA results showed that JAK/STAT signaling pathway, MAPK signaling pathway, and Wnt receptor signaling pathway were enriched in the AS group. Additionally, a majority of the genes were also enriched in these pathways in the high STAT3 expression group. JAK/STAT, Wnt receptor, and MAPK signaling pathway were strongly associated with osteoblast differentiation and bone formation. These results demonstrated STAT3 may be involved in osteoblast differentiation and bone formation in AS patients via JAK/STAT, Wnt receptor, and MAPK signaling pathways.

In this study, STAT3 was significantly positively correlated with the xCell scores of NKT cells and negatively correlated with the xCell scores of Th1 cells. STAT3 plays a key role in activating NKT cells by targeting its downstream MHC class I-related chain (MIC) [22]. The activation of STAT3 may suppresses Th1 response during tuberculosis infection [23]. TIRC7 inhibits Th1 cells by upregulating the expression of CTLA‑4 and STAT3 in mice with acute graft‑versus‑host disease [24]. These results demonstrated that the upregulating of STAT3 may activate NKT cells and suppresses Th1 response. NKT cells could inhibit osteoclastogenesis by secreting large amounts IL‐4, which is known to be a suppressive factor for osteoclastogenesis [25]. Th1 cells has biphasic effects in the procedure of osteoclastogenesis by secreting IFN-γ and TNF-α, which can promote or inhibit the differentiation of osteoclasts [26]. As for STAT3, which was upregulated in AS patients, it can directly or indirectly induce the differentiation of osteoclasts.

SPI1 (PU.1) encodes an ETS-domain transcription factor that activates gene expression during myeloid and B-lymphoid cell development. Diseases associated with SPI1 include inflammatory diarrhea and erythroleukemia [27–32]. Among its related pathways are glucocorticoid receptor regulatory network and IL-4 signaling and its primary biological effects in different immune cell types. SPI1 have been previously shown to play important roles in the differentiation and function of osteoblasts [33]. SPI1 regulates dental pulp stem cell osteoblastic differentiation through noggin [34]. Elevated SPI1 promotes BMP2 transcription and further accumulates vascular calcification [35]. The GSEA results also showed that JAK/STAT signaling pathway, MAPK signaling pathway, and Wnt receptor signaling pathway were enriched in the high SPI1 expression group. As we just mentioned, these pathways were strongly associated with osteoblast differentiation and bone formation. There are also many studies demonstrated that up-regulated PU.1 may lead to osteoporosis by promoting osteoclast differentiation [36–39]. PU.1 may cause osteoclast differentiation and osteogenesis simultaneously in AS.

In this study, SPI1 was also significantly positively correlated with the xCell scores of NKT cells and negatively correlated with the xCell scores of Th1 cells. PU.1 involved in the transcriptional regulation of CD1D1, which plays an important role in the development and function of NKT cells regulating innate and adaptive immunity, by binding to the CD1D1 promoter [40]. Besides, PU.1 also involved in the differentiation of TH1 cells [41–45]. PU.1 may also play a role in heterotopic ossification and osteoporosis in AS by regulating NKT and TH1 cells differentiation.

In the present study, we demonstrated STAT3 and SPI1 may be involved in osteoblast differentiation and bone formation in AS patients via JAK/STAT, Wnt receptor, and MAPK signaling pathways. Besides, STAT3 and SPI1 may also affect heterotopic ossification and osteoporosis in AS through regulating NKT and TH1 cells differentiation. However, there are some limitations that exist in this study. We just test NKT cells and Th1 cells in peripheral blood by flow cytometry, which made our research not strict adequate and the experimental conclusion was not sufficient enough.

## Conclusion

According to our analysis of the microarray datasets related to AS, STAT3 and SPI1 were identified as the hub genes, could work as diagnostic markers of AS. STAT3 and SPI1 may be involved in the NKT and Th1 cells activation, proliferation and differentiation in AS. Moreover, STAT3 and SPI1 may be involved in osteoblast differentiation and bone formation in AS patients via JAK/STAT, Wnt receptor, and MAPK signaling pathways or through regulating NKT and TH1 cells differentiation.

## Materials and methods

### GEO datasets

Gene expression profile data were obtained from the gene expression omnibus (GEO) (www.ncbi.nlm.nih.gov/gds), a public repository at the National Center of Biotechnology Information that contains high-throughput gene expression datasets. GSE73754, which contains 52 AS and 20 healthy controls (HCs), was downloaded for further analysis.

### Weighted Correlation Network Analysis (WGCNA) network construction and module identification

We used the WGCNA (version 1.70-3) R package to construct the co-expression network. Before WGCNA, samples with obvious outlier were excluded and genes with low quality were screened and filtered. The R function pick soft-threshold was used to calculate the soft thresholding power β, to which co-expression similarity is raised to calculate adjacency. We performed hierarchical clustering to identify modules, each containing at least 50 genes. Next, we calculated the eigengene, hierarchically clustered the modules, and merged similar modules (abline = 0.25). Gene significance and module membership were calculated to relate modules to clinical characteristics. The corresponding module gene information was extracted for further analysis.

### KEGG/GO enrichment analyses and hub genes identification

The genes in the red model were performed enrichment analyses using the “clusterProfiler” package (version 3.16.0) and then visualized using the ‘ggplot2’ package (version 3.3.0) in R studio. Gene ontology (GO) and Kyoto Encyclopedia of Genes and Genomes (KEGG) terms with adjusted *p*-value < 0.05 were considered significantly enriched. The protein–protein interaction (PPI) network was predicted using the search tool for the retrieval of interacting genes (STRING; http://string-db.org) (version 11.0) online database and was visualized using Cytoscape (version 3.6.1). The cut-off value for STRING analysis is 0.04. We used the Cytoscape plug-in APP “cytoHubba” for hub genes identification and the top 5 nodes ranked by “Degree”, “Betweenness”, and “Closeness” were enrolled in gene selection. And the overlap genes were further evaluated their diagnosis ability and relationship with clinical characteristics.

### Immune and stromal cells analyzed

The ‘xCell’ package was used to calculate the cell type enrichment score (xCell scores) based on the gene expression data. Notably, the xCell tool provides 64 cell types, including lymphoid, myeloid, stromal cells, stem cells, and other cells. The difference between each cell subtype between AS and HCs was compared using the t-test method. The cut-off values for the cell analyses were *p* value < 0.05.

### Correlation test between hub genes and immune cells

Pearson correlation analysis between SPI1 and STAT3, and the xCell score of the significant difference cell types was performed with the ‘corrplot’ package (version 0.84). Pearson correlation coefficient > 0.4 and *p* value < 0.05 were considered as significantly correlated.

### Flow cytometry analysis

A total of 24 patients included 12 AS patients and 12 lumbar spinal stenosis patients as a control group were enrolled in this study (Table 1). All patients with AS were newly diagnosed without medicine treatment and the patients with co-diagnosis were excluded. All the AS patients were diagnosed according to the modified New York criteria (Evaluation of diagnostic criteria for ankylosing spondylitis. A proposal for modification of the New York criteria) [13]. Subjects volunteering for the study had signed informed consent forms. In addition, our study was approved by the Ethics Department of the First Affiliated Hospital of Guangxi Medical University (KE-E-044) and conformed to the World Medical Association Declaration of Helsinki.

**Table 1:** Baseline characteristics of the patients

Characteristics	AS	HC	Overall	*P*-value
	(N = 12)	(N = 12)	(N = 24)	
*Age*				
Mean (SD)	40.4(7.25)	54.9(10.7)	47.7(11.6)	< 0.001
Median [Min, Max]	40.0[28.0,52.0]	55.0[34.0,68.0]	47.0[28.0,68.0]	
*Sex*				
Female	11(91.7%)	7(58.3%)	18(75.0%)	0.157
Male	1(8.3%)	5(41.7%)	6(25.0%)	
*BMI*				
Mean (SD)	20.7(2.46)	21.8(3.74)	21.2(3.15)	0.395
Median [Min, Max]	21.2[16.0,24.7]	21.9[15.9,27.7]	21.4[15.9,27.7]	
*WBC*				
Mean (SD)	8.78(2.68)	7.31(2.38)	8.04(2.59)	0.168
Median [Min, Max]	9.04[5.05,13.6]	6.23[5.01,12.4]	7.68[5.01,13.6]	
*Neu%*				
Mean (SD)	0.718(0.0856)	0.649(0.113)	0.683(0.104)	0.109
Median [Min, Max]	0.720[0.591,0.831]	0.613[0.538,0.905]	0.683[0.538,0.905]	
*Lym%*				
Mean (SD)	0.188(0.067)	0.247(0.105)	0.218(0.091)	0.124
Median [Min, Max]	0.187[0.103,0.296]	0.254[0.062,0.380]	0.207[0.062,0.380]	
*Mon%*				
Mean (SD)	0.072(0.023)	0.077(0.029)	0.075(0.026)	0.662
Median [Min, Max]	0.066[0.040,0.115]	0.078[0.028,0.123]	0.074[0.028,0.123]	
*ESR*				
Mean (SD)	30.4(19.8)	18.2(28.1)	24.3(24.6)	0.232
Median [Min, Max]	29.0[5.00,70.0]	6.50[3.00,98.0]	14.0[3.00,98.0]	
*CRP*				
< 10	1 (8.3%)	7 (58.3%)	8 (33.3%)	0.03
> 10.00	11 (91.7%)	5 (41.7%)	16 (66.7%)	
*hsCRP*				
< 0.8	1 (8.3%)	8 (66.7%)	9 (37.5%)	0.004
> 10.00	11 (91.7%)	3 (25.0%)	14 (58.3%)	
> 10	0 (0%)	1 (8.3%)	1 (4.2%)	
*HLA-B27*				
Positive ( +)	12 (100%)	0 (0%)	12 (50.0%)	< 0.001
−	0 (0%)	12 (100%)	12 (50.0%)	
*Diagnosis*				
AS	12 (100%)	0 (0%)	12 (50.0%)	< 0.001
LSS	0 (0%)	12 (100%)	12 (50.0%)	

AS: Ankylosing Spondylitis; LSS: Lumbar Spinal Stenosis

Blood samples were taken in the Department of Spine and Osteopathy Ward, the First Affiliated Hospital of Guangxi Medical University. After peripheral venous blood sampling, 5-mL samples preserved with Ethylene Diamine Tetraacetic Acid (EDTA) anticoagulant tubes were used to detect immune cells. The samples were stored at room temperature (20 ~ 30 °C) for no more than six-hour before handling. The blood sample were divided it into two tubes for flow cytometry analysis of NKT and Th1 cells respectively. Fluorescein isothiocyanate (FITC)-CD3, FITC-CD4, phycoerythrin (PE)-CD56, allophycocyanin (APC)-IFN-γ, which were purchased from eBioscience (SanDiego, CA, USA), were used for phenotypic analysis on PBMCs. The data were compensated and analyzed according to the following gating strategy: The NKT and Th1 cells were separated by setting an appropriate gate. NKT cells were doublestained with CD3-FITC and CD56-PE, and Th1 cells were doublestained with CD4-FITC and IFNG-APC. Stained cells were analyzed with BD FACSVerse flow cytometer and results were analyzed was FlowJo VX software. We performed statistical analysis and visualization of the percentage of the two cell types using GraphPad Prism 8.

### Gene set enrichment analysis (GSEA)

GSEA (V4.0.3), which is a computational method that determines whether an a priori defined set of genes shows statistically significant concordant differences between two biological states [14], was performed to investigate the potential biological characteristic between high-NKT/Th1 xCell score and low-NKT/Th1 xCell score based on immune-related gene ontology gene sets downloaded from the Molecular Signatures Database [15]. We only investigated c2 (c2.cp.kegg.v7.2.symbols.gmt) gene sets in the current study. The significant cutoff value was defined as the false discovery rate (FDR < 0.25) and the normal *p* value < 0.05.

### Statistical analysis

A student’s t-test was used to compare the means of the continuous variables between two groups. The performance of the factors and nomogram was assessed using the ROC curves (“pROC” package). A two-sided probability value less than 0.05 was considered to be statistically significant for all analyses.

## Supplementary Information

**Additional file 1**. Cell types in the microenvironment of AS. (A, B, C, D, E) xCell score of sixty-four cell types in GSE73754 were grouped into five groups: lymphoid, myeloid, stem, stromal, and other cells.

## Data Availability

The GEO datasets (GEO ID: GSE73754) generated and/or analyzed during the current study are available in the GEO database (www. ncbi.nlm.nih.gov/gds). And all data and materials for this study shall be availed whenever requested by the editorial team, reviewers, and other users. The data set can be accessed by sending a request to 15,676,790,870@163.com.

## Abbreviations

TME: Tumor microenvironment
TIICs: Tumor-infiltrating immune cell
GSEA: Gene set enrichment analysis
GO: Gene ontology
KEGG: Kyoto encyclopedia of genes and genomes
DEGs: Differentially expressed genes
AS: Ankylosing spondylitis
IL2RB: Interleukin 2 receptor subunit beta
NKT: Natural killer T cells
STAT3: Signal transducer and activator of transcription 3
SPI1: Spi-1 proto-oncogene
