# Supplementary figures and images for: STAT3 and SPI1, may lead to the immune system dysregulation and heterotopic ossification in ankylosing spondylitis
Source: BMC Immunol. 2022 Jan 22;23:3. doi: 10.1186/s12865-022-00476-6 (PMC8783415; doi:10.1186/s12865-022-00476-6)

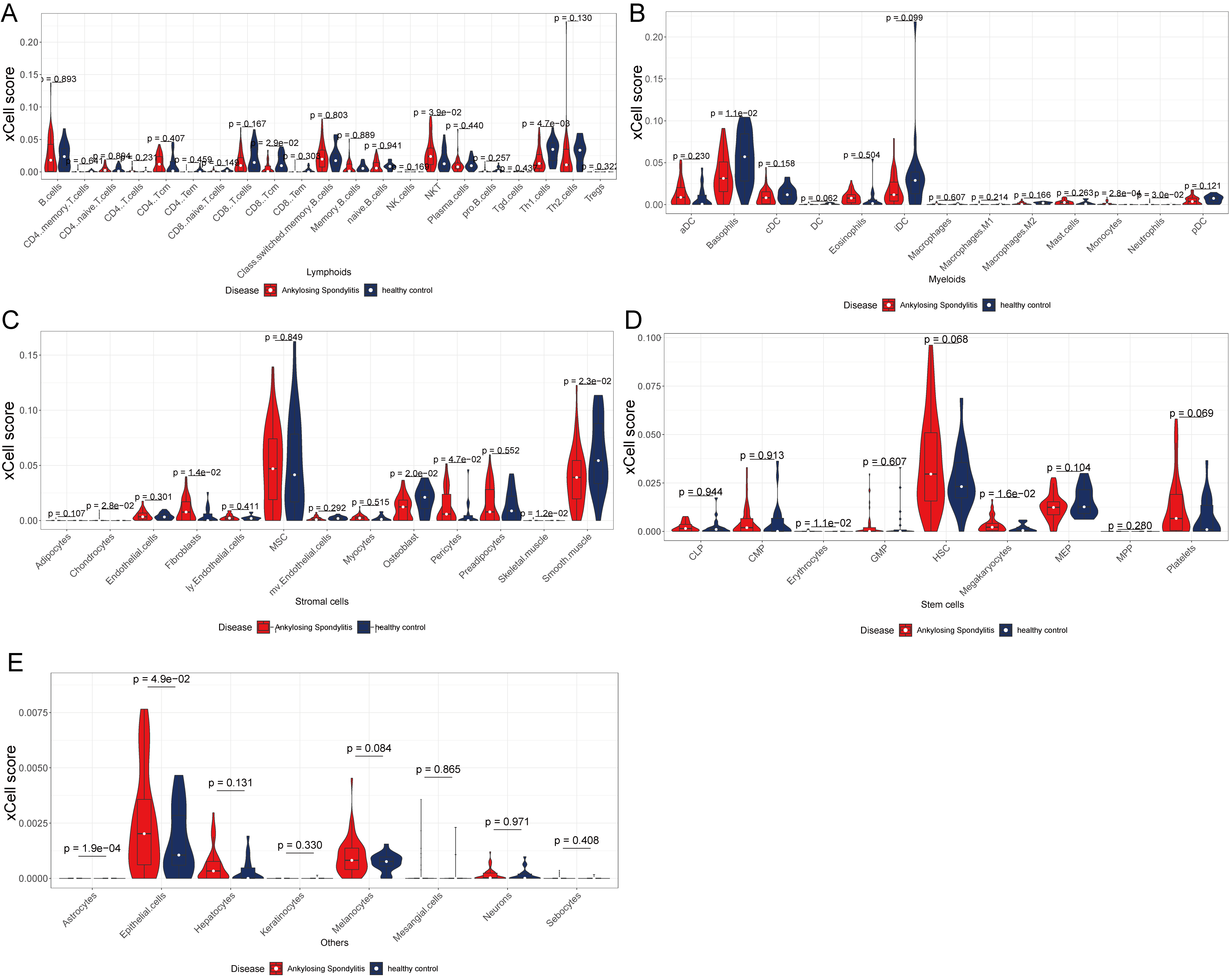

Supplement: Supplementary file 1 — Additional file 1. Cell types in the microenvironment of AS. (A, B, C, D, E) xCell score of sixty-four cell types in GSE73754 were grouped into five groups: lymphoid, myeloid, stem, stromal, and other cells. [file 12865_2022_476_MOESM1_ESM.tif]
